# Metaheuristic Optimization of Random Forest for Predicting Punch Shear Strength of FRP-Reinforced Concrete Beams

**DOI:** 10.3390/ma16114034

**Published:** 2023-05-28

**Authors:** Peixi Yang, Chuanqi Li, Yingui Qiu, Shuai Huang, Jian Zhou

**Affiliations:** 1 School of Resources and Safety Engineering, Central South University, Changsha 410083, China; 215511049@csu.edu.cn (P.Y.); 225501014@csu.edu.cn (Y.Q.); 205511038@csu.edu.cn (S.H.); 2 Laboratory 3SR, CNRS UMR 5521, Grenoble Alpes University, 38000 Grenoble, France

**Keywords:** reinforced concrete, punching shear strength, random forest, ant lion optimizer, moth flame optimizer, salp swarm algorithm

## Abstract

Predicting the punching shear strength (*PSS*) of fiber-reinforced polymer reinforced concrete (FRP-RC) beams is a critical task in the design and assessment of reinforced concrete structures. This study utilized three meta-heuristic optimization algorithms, namely ant lion optimizer (ALO), moth flame optimizer (MFO), and salp swarm algorithm (SSA), to select the optimal hyperparameters of the random forest (RF) model for predicting the punching shear strength (*PSS*) of FRP-RC beams. Seven features of FRP-RC beams were considered as inputs parameters, including types of column section (*TCS*), cross-sectional area of the column (*CAC*), slab’s effective depth (*SED*), span–depth ratio (*SDR*), compressive strength of concrete (*CSC*), yield strength of reinforcement (*YSR*), and reinforcement ratio (*RR*). The results indicate that the ALO-RF model with a population size of 100 has the best prediction performance among all models, with MAE of 25.0525, MAPE of 6.5696, R^2^ of 0.9820, and RMSE of 59.9677 in the training phase, and MAE of 52.5601, MAPE of 15.5083, R^2^ of 0.941, and RMSE of 101.6494 in the testing phase. The slab’s effective depth (*SED*) has the largest contribution to predicting the *PSS*, which means that adjusting *SED* can effectively control the PSS. Furthermore, the hybrid machine learning model optimized by metaheuristic algorithms outperforms traditional models in terms of prediction accuracy and error control.

## 1. Introduction

Fiber-reinforced polymers (FRPs) are increasingly used as a substitute for traditional steel reinforcement in civil engineering due to their lightweight, corrosion-resistant, and high-strength properties [1]. Composite structures of fiber-reinforced polymers (FRP) and concrete contribute to enhance the resistance and stiffness of structures while reducing the susceptibility of concrete to external corrosion [2]. Consequently, such structures are suitable for applications in retrofitting, rehabilitation, and repair projects [3,4,5]. As a critical structural element in the building construction, the performance assessment of FRP-reinforced concrete (RC) beams became a research hotspot and difficulty [6,7,8,9,10]. The composite structure of FRP-RC has the potential to improve the load-bearing capacity, durability, and sustainability of beams, making it an attractive option for a variety of construction applications. However, the shear strength of FRP-RC beams plays an important role in structural design, but it is difficult to calculate accurately.

The shear strength of FRP-RC beams can be calculated using experimental methods [8,11,12,13], numerical simulations [6,14,15,16], and artificial intelligence (AI) prediction methods [17,18,19]. Experimental analysis serves as an intuitive method for evaluating the performance of beams. However, using traditional mechanical methods to explain the shear behavior of FRP-RC beams is a challenging task [20]. This complexity significantly increases the costs associated with human resources, materials, and time. Numerical simulation methods demonstrated efficacy in analyzing the shear strength of FRP-RC beams. Nevertheless, the required loading conditions of the beams must be assumed for the practical applications, and obtaining valuable experimental data for modeling purposes is generally difficult. Furthermore, the mathematical structure of the models tends to be rather extensive, which complicates the model-building process [19]. Consequently, there is a need for a more efficient and accurate approach to predict the shear strength of FRP-RC beams.

With the continuous advancement of computer technology, artificial intelligence (AI) techniques represented by machine learning (ML) models are increasingly applied in the field of civil engineering, particularly in the prediction of the performance of RC beams [21,22,23,24,25,26,27,28,29,30]. Mansour et al. [31] utilized the artificial neural network (ANN) model to predict the shear performance of RC beams. The prediction results show that the ANN model can significantly improve the prediction accuracy compared to the empirical formula method. However, the development of the ANN model requires the determination and definition of its structure and parameters, which is time-consuming [32]. Abuodeh et al. [19] employed the resilient back-propagating neural network (RBPNN) model to explore the enhancement effects of FRP on the shear stress of RC beams. They analyzed the impact of various factors on the shear capacity of FRP-RC beams. Kaloop et al. [33] obtained promising results in predicting the shear strength of RC deep beams using the support vector regression (SVR) optimized by the African vultures optimization algorithm (AVOA), achieving a maximum error of ±3.39%. However, the performance of the BPNN model is heavily reliant on the quality and quantity of input data, and insufficient or unsuitable data potentially will lead to overfitting [34]. The SVR model is sensitive to the selection of the kernel function and hyperparameter values [35], and the prediction of the shear strength of FRP-RC beams is a complex nonlinear problem, which makes it difficult to select an appropriate kernel function.

Random forest (RF) is a type of ensemble ML model proposed by Breiman [36], which combines multiple decision trees to improve robustness and prevent overfitting. The feature importance scores provided by the RF model further improve the interpretability of the model. Owing to its exceptional performance, RF was extensively employed in predicting the mechanical properties of reinforced concrete (RC) in construction engineering [37,38,39,40,41]. Mohammed et al. [42] compared the performance of support vector machines (SVM) and RF models for predicting the shear strength of RC beams and found that the RF model has a better prediction accuracy than the SVM model. Zhang et al. [32] compared the prediction performance of the BPNN and RF models for estimating the shear strength of RC beams with and without stirrups. The results show that the RF model significantly outperformed the BPNN model. Feng et al. [20] conducted a comparison of various ML models and discovered that the RF model has strong performance for predicting the lateral strength of reinforced concrete (RC) walls, effectively estimating the shear strength of RC materials. In light of the aforementioned literature, it can be concluded that the RF model exhibits outstanding performance for predicting the shear strength of FRP-RC beams. Metaheuristic optimization algorithms represent a category of optimization techniques inspired by natural processes, frequently employed to determine the optimal combination of hyperparameters for improving the performance of ML models [43], and their efficiency in searching complex search spaces was applied in civil engineering [44]. Moayedi et al. [45] used the ant lion optimizer (ALO) to predict the soil shear strength, significantly improving the prediction accuracy of the ANN model. Huang et al. [46] used the moth–flame optimization (MFO) to improve the prediction accuracy of damage in concrete beams. Khajehzadeh et al. [47] used the salp swarm algorithm (SSA) to optimize the design of steel-reinforced concrete cantilever retaining walls and shallow foundation engineering structures. It is evident that the application of these metaheuristic algorithms can effectively enhance the predictive performance of the ML method in the field of structural engineering.

This paper aims to predict the punching shear strength (PSS) of FRP-RC beams using the RF model optimized by three metaheuristic algorithms (ALO, MFO, and SSA). The predictive performance of these hybrid models was compared and the best one was selected to predict the PSS of FRP-RC beams. The paper is organized as follows: Section 2 presents the three metaheuristic algorithms coupled with the RF model. Then, the preparation of the punching shear strength database for FRP-RC beams, the data processing, and the model evaluation metrics and interpretation methods are described in Section 3. Section 4 presents the predictive performance of the models and compares their performance. In this Section, feature importance analysis is adopted for the interpretation of model prediction performance. Finally, Section 5 summarizes the findings of the study and provides recommendations for future research.

## 2. Methodologies

### 2.1. Random Forest

**Figure 1:**
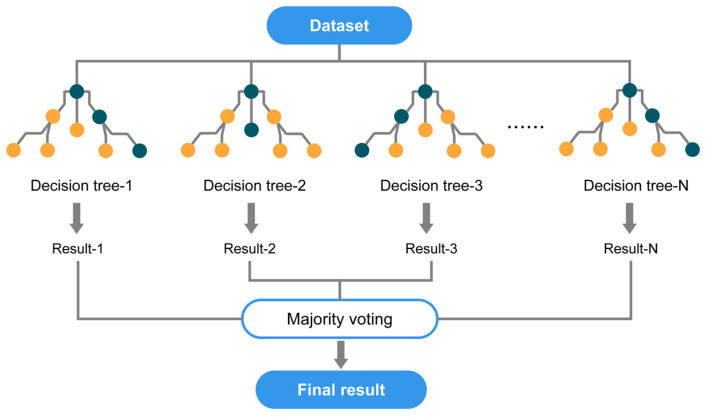
Algorithm flow of random forest.

The random forest algorithm is a versatile and widely used ensemble learning technique in the field of machine learning and data science. It was first introduced by Leo Breiman in 2001 [36], combining the strengths of multiple decision tree models to improve overall accuracy and robustness [48], as shown in Figure 1. The key principle behind the random forest algorithm is the creation of numerous decision trees during the training phase, which then vote collectively to make a final prediction:

(a) Bagging: The algorithm employs a technique known as bootstrap aggregating, or “bagging.” This process involves constructing multiple decision trees by randomly sampling the training data with replacement, which results in a diverse set of trees;
(b) Feature randomness: At each node of the decision tree, the algorithm selects a random subset of features rather than using all features to make a split. This approach introduces additional randomness and further diversifies the individual trees, making the final model more robust;
(c) Voting mechanism: During the prediction phase, each tree in the random forest contributes its independent decision, and the final prediction is determined by a majority vote among the trees. This ensemble approach reduces the risk of overfitting and improves the generalization capabilities of the model.

The final prediction of the RF model is obtained by averaging the predictions of all the trees as follows:

### 2.2. The Ant Lion Optimizer

The ant lion optimizer (ALO) is a meta-heuristic optimization algorithm proposed by Mirjalili [49] and inspired by the hunting behavior of ant lions. The ALO algorithm showed good performance in solving the complex optimization problems with high efficiency and accuracy. The algorithm involves the creation of an ant lion-inspired search space that consists of a central pit and surrounding terrain. In the ALO algorithm, the movement of the ant represents the exploration of the search space, while the lion represents the exploitation of the search space. The algorithm also includes a step of updating the pheromone trail, which enhances the convergence rate of the algorithm.

The free movement taken by ants in space while searching for food in nature can be expressed by the following equation:

(1) $$\begin{array}{l} X(i)=[0,cs(2r(i_{1})-1),cs(2r(i_{2})-1),\ldots \ldots ,cs(2r(i_{n})-1)]\\ r(i)=\{\begin{array}{c} 1,\;if\;rand>0.5\\ 0,\;if\;rand\leq 0.5 \end{array} \end{array}$$

where X denotes the position of the current ant individual, *cs* denotes the calculated cumulative sum, *i* denotes the number of iterations, *n* denotes the maximum number of iterations, and rand denotes a random number of [0, 1].

To simulate the hunting ability of ant lions, a roulette strategy was used to make it easier for the well-adapted ant lions to capture ants. When an ant approaches the trap of an ant lion, the random movement range of the ants is limited to a particular trap (the ants move randomly in a hypersphere layer). When an ant falls within the trap, the ant lion will spray sand at the ant, causing the ant to move closer and closer to the ant lion in the trap. Eventually, the ant lion captures the ants that fall into the trap and updates its position to the latest position of the captured ants in order to be able to capture them better, and this behavior is expressed in the mathematical model as follows:

(2) $$Antlion_{j}^{s}=Ant_{k}^{s}\;,\;if\;f(Ant_{k}^{s})>f(Antlion_{j}^{s})$$

where $Antlion_{j}^{s}$ represents the position of the *j*-th ant lion at the *s*-th iteration and $Ant_{k}^{s}$ represents the position of the *k*-th ant at the *s*-th iteration.

**Figure 2:**
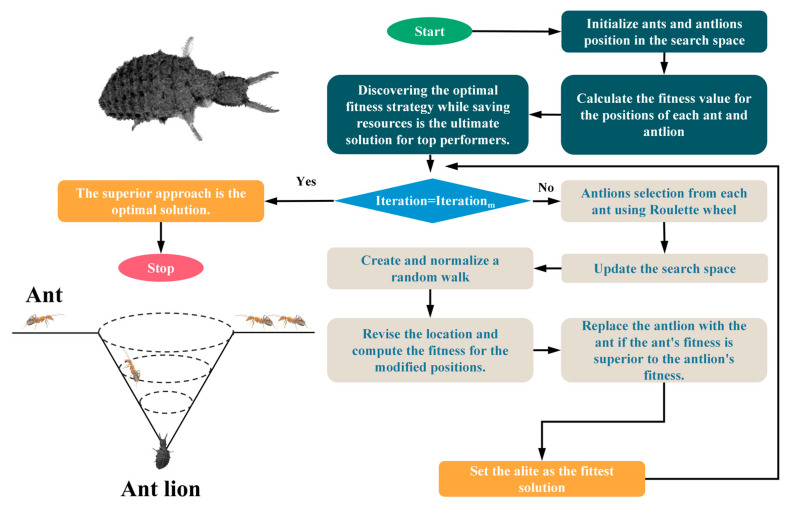
Flow chart of ant lion optimization algorithm.

In each iteration, the information of the best one is retained and affects the walking range of the ants in each future iteration, and this elite strategy maintains the best solution obtained at any stage of the optimization process. The flow of the ALO algorithm described above is shown in Figure 2:

### 2.3. The Moth–Flame Optimization

The moth–flame optimization (MFO) algorithm is a novel intelligent optimization algorithm proposed by Seyedali Mirjalili and his colleagues in 2015 [50]. The algorithm is inspired by the unique navigation mechanism of moths during nocturnal flights, in which they use a lateral positioning mechanism to maintain a fixed angle relative to the moon, enabling straight flight using approximate parallel light from the distant moon. The MFO algorithm uses a mathematical model that incorporates concepts such as logarithmic spirals and a distance-based sorting mechanism to guide the moths towards the global optimum.

In the MFO algorithm, moths update their positions in space only with respect to a unique flame that corresponds to them, thus avoiding the algorithm being trapped in local optima. Moths continually move and search within the search space, with the position of the flame being the target position for the corresponding moth. The behavior of moths flying towards the flame can be expressed mathematically as follows:

(3) $$M_{j}=R(M_{j},F_{k})$$

where *M_j_* represents the *j*-th moth and *F_k_* represents the *k*-th flame; *R* is the spiral function.

**Figure 3:**
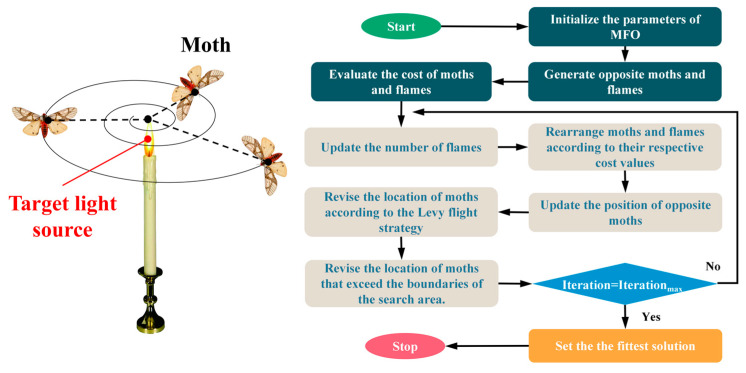
Flow chart of the moth–flame optimization.

Moths fly around specific flames in the MFO algorithm. When the fitness of any moth is higher than that of any flame, the position of the flame is updated accordingly. The flames also adaptively decrease during the iterative process, maintaining a balance between the global search capability and the local exploitation capability of the algorithm. The relevant mathematical formulae are presented as follows:

(4) $$f_{no}=rand(N_{f}-t\times \frac{N_{f}-1}{T})$$

where *N_f_* denotes the maximum number of flames, *t* denotes the current number of iterations, and *T* denotes the maximum number of iterations. The algorithmic flow of MFO described above can be represented in Figure 3:

### 2.4. The Salp Swarm Algorithm

The salp swarm algorithm (SSA) is a bio-inspired optimization algorithm proposed by Mirjalili et al. [51] in 2014. SSA is inspired by the swimming behavior of salps, which are barrel-shaped planktonic tunicates that move by contracting and expanding their bodies.

During the foraging and movement in the ocean, salps often follow each other in a chain-like manner, with individuals connected head-to-tail. In such a “chain” group, there are leaders and followers, with the leaders responsible for tracking food and each follower only influenced by the salp in front of it. The position change of the leader during the foraging process of the salp population can be expressed by using the following equation:

(5) $$S_{1}=\{\begin{array}{l} G+h_{1}(ub-lb)h_{2}+lb),\;h_{3}\geq 0.5\\ G-h_{1}(ub-lb)h_{2}+lb),\;h_{3}<0.5 \end{array}$$

where *S*_1_ represents the location of the first salp leader in the search space, *G* denotes the location where the food is located, *ub* and *lb* denote the upper and lower bounds of the search space, respectively, *h*_1_ represents the convergence factor in the optimization algorithm, and *h*_2_ and *h*_3_ represent the random number of [0, 1].

**Figure 4:**
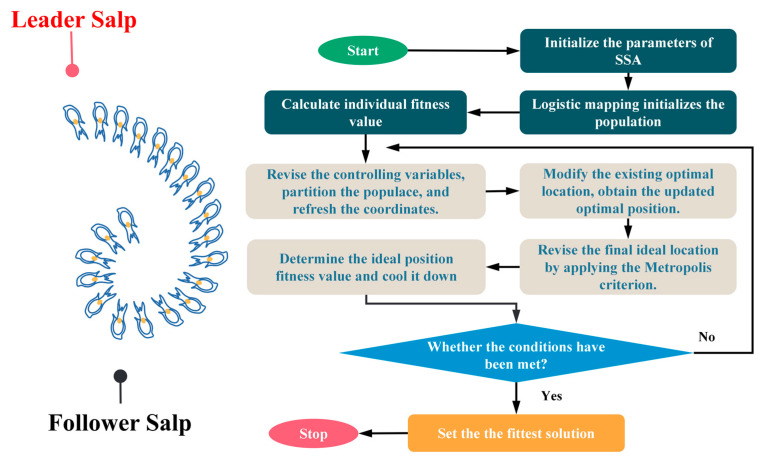
Flow chart of the salp swarm algorithm.

The follower moves with the change in position of the previous salp, so that the position of the follower changes as shown below:

(6) $$S^{j^{\prime }}=\frac{S^{j}-S^{j-1}}{2}$$

where *S^j^* and *S^j^*^−1^ denote the two adjacent salp positions before the update and *S^j^*′ denotes the updated salp position. The above SSA algorithm flow can be expressed in Figure 4:

## 3. Data Manipulation and Performance Evaluation

### 3.1. Punching Shear Strength of FRP-RC Beams

The PSS is a crucial mechanical characteristic of FRP-RC beams [52,53,54,55,56], which refers to the ability of the beam to resist the shear force at the point of contact with a concentrated load, such as a column or a pedestal. The punching shear failure mode can result in catastrophic failure of the beam and poses a significant challenge for the design and construction of FRP-RC structures [57].

The PSS of FRP-RC (fiber-reinforced polymer-reinforced concrete) beams is typically influenced by numerous factors. Different column section types can affect the force distribution and overall stability of the structure, while the size of the column’s cross-sectional area also has an impact on the distribution of loads. The effective depth of the slab, usually defined as the distance from the extreme compression fiber to the centroid of the tensile reinforcement, plays a significant role in punching shear strength; a larger effective depth generally results in a higher moment of inertia, thereby leading to increased shear strength. On the other hand, a lower span–depth ratio typically corresponds to greater punching shear strength. In terms of material properties, the compressive strength of concrete, the yield strength of reinforcement, and the reinforcement ratio all contribute to the punching shear strength of FRP-RC beams. Understanding the interplay between these factors is essential for optimizing the design and overall performance of such structures.

ML models emerged as promising tools for predicting the PSS of FRP-RC beams [58,59]. By training an ML model with a large dataset of experimental results, the model can learn to identify the underlying patterns and relationships between the input parameters and the punching shear strength. The ML model can provide a more accurate and efficient prediction compared to traditional analytical methods that rely on empirical equations or simplifying assumptions.

### 3.2. Construct Database

This study aimed to create a comprehensive database of punching shear strength (PSS) for FRP-RC columns by compiling data from 26 published works [60,61,62,63,64,65,66,67,68,69,70,71,72,73,74,75,76,77,78,79,80,81,82,83,84,85]. The database contains information on several parameters, which can be categorized as follows: (1) geometric parameters, including the type of column section (TCS) represented by 1, 2, and 3 for circular, rectangular, and square columns, respectively, cross-sectional area of the column (CAC), slab’s effective depth (SED), and span–depth ratio (SDR); (2) concrete strength information, including the compressive strength of concrete (CSC); and (3) steel reinforcement information, including the yield strength of reinforcement (YSR) and reinforcement ratio (RR). The output parameter is the PSS of the beams. The values are presented in Table 1, which includes the units and statistical information. Some of the data from the FRP-RC PSS database are presented in Table 2.

### 3.3. Parameter Correlation Analysis

**Figure 5:**
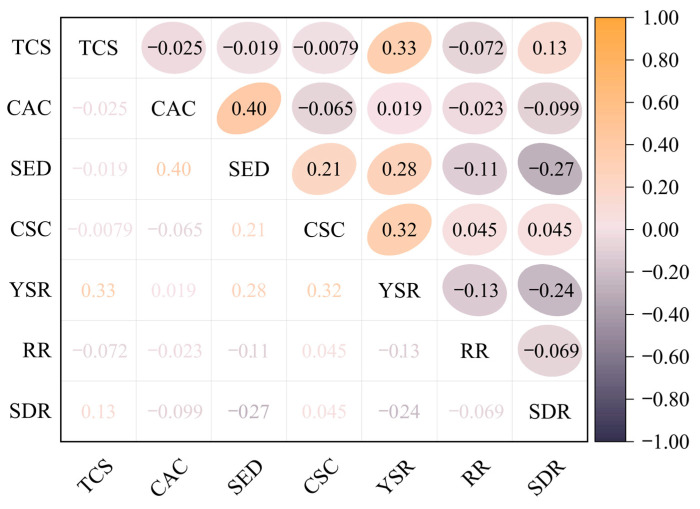
Pearson correlation coefficient analysis of the characteristic parameters of FRP-RC beams.

Performing correlation analysis on parameters before establishing a ML model helps to identify the strength and direction of the relationships between the input variables [86]. The Pearson correlation coefficient is a statistical measure that evaluates the linear relationship between two continuous variables. It measures the degree to which the variables are associated and ranges from −1 to +1, with −1 indicating a perfectly negative linear relationship, 0 indicating no linear relationship, and +1 indicating a perfectly positive linear relationship [87]. The Pearson correlation coefficient analysis is commonly used in ML models to analyze the correlation between input parameters [88,89,90,91,92,93]. If the correlation coefficient between input parameters is high (generally above 0.8), it is necessary to consider selecting or discarding certain parameters to avoid data redundancy [94]. In this study, Pearson correlation coefficient analysis was performed on the characteristic parameters of seven parameters of FRP-RC beams, and the results are shown in Figure 5. It can be observed that the correlation coefficients between SDR, RR, YSR, SED, CAC, TCS, and CSC are all at a low level, indicating that there is no strong correlation between these parameters of the FRP-RC beams. Therefore, they can all be used as input parameters for generating the ML model without redundancy or increasing the amount of ineffective calculations.

### 3.4. Evaluation Indicators

In the construction of various machine learning (ML) models [95,96], the selection of appropriate evaluation metrics plays a critical role in assessing the reliability and accuracy of the models. For solving the regression problems, the mean absolute error (MAE), the mean absolute percentage error (MAPE), the coefficient of determination (R^2^), and the root mean square error (RMSE) are widely used as evaluation metrics for regression models [93,97,98,99,100]. The MAE measures the average absolute difference between the predicted values and the measured values, which provides a measure of the accuracy of the model in predicting the target variable; the MAPE measures the average percentage difference between the predicted values and the measured values; and the R^2^ is a statistical measure that represents the proportion of the variance in the target variable that can be explained by the independent variables in the model. It ranges from 0 to 1, with higher values indicating a better fit between the model and the data; the RMSE measures the root mean squared difference between the predicted values and the measured values. In this study, MAE, MAPE, R^2^, and RMSE are selected as evaluation metrics for the ML model. Their definitions and calculation formulas are presented in Table 3.

### 3.5. Hybrid Models

**Figure 6:**
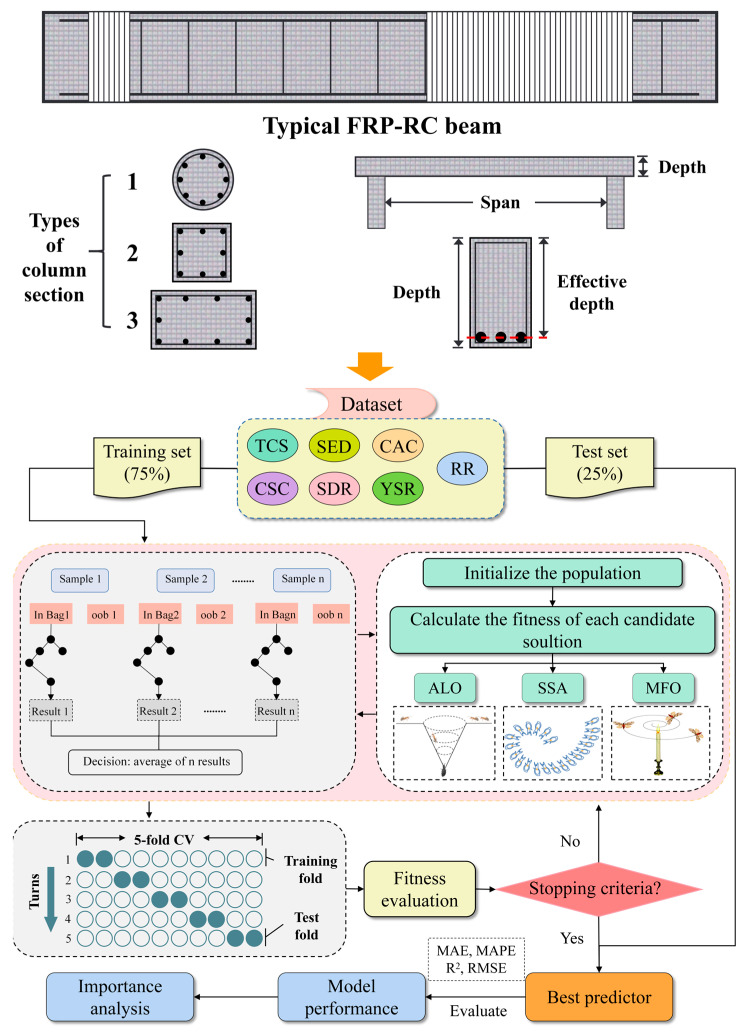
General flow chart of the algorithm.

The database used in this study consists of 607 sets of data for FRP-RC beams. As shown in Figure 6, 75% of 607 sets of data were used as the training set, and the remaining 25% were used as the test set. In addition, all data were normalized to the range of −1 to 1. Cross-validation (CV) is a common technique used in machine learning to evaluate the performance of a model for estimating the performance of a model on new data and can help prevent the model from overfitting to the training data [101]. In this study, a five-fold CV is used in the training set where the dataset is divided into five equally sized subsets, and the process is repeated five times, with each subset used once as a validation set. In addition, the average RMSE value generated from five-fold CV was used as the fitness value [102].

In the RF model, the hyperparameters that need to be optimized are usually the number of trees and the maximum depth of trees [103]. Population size in machine learning refers to the number of individuals or data points within a population or dataset that are used for training and optimization purposes. In evolutionary algorithms and genetic programming, population size determines the diversity and exploration–exploitation trade-off during the search process [43]. Therefore, the population size in this study was set at 10, 50, 100, and 200, and the other parameter settings are presented in Table 4. Then, this study employed three types of meta-heuristic optimization algorithms, namely ALO, MFO, and SSA, to optimize the hyperparameters of the RF model. Subsequently, four evaluation indicators were used to assess the PSS prediction performance of the three hybrid models (ALO-RF, MFO-RF, and SSA-RF). Finally, the importance of each input parameter was analyzed to further enhance the interpretability of the model. The computational flowchart of the above models is shown in Figure 6.

## 4. Results and Discussion

### 4.1. Model Validity Judgment

**Figure 7:**
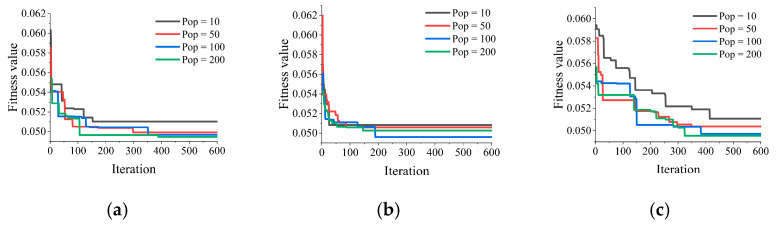
Variation curve of fitness value of hybrid model. (**a**) ALO-RF, (**b**) MFO-RF, and (**c**) SSA-RF.

The RMSE is a widely used evaluation metric in machine learning because it provides a measure of the overall difference between the predicted values and the actual values, taking into account both the magnitude and direction of the errors. The aforementioned parameters render the PSS an appropriate selection for use as the fitness or objective function in optimization algorithms, which aim to identify the optimal set of model parameters that minimize the discrepancy between the predicted and actual values [104]. In this study, three different optimization algorithms, namely ALO, MFO, and SSA, were employed to optimize the hyperparameter combinations in the RF model for predicting the punching shear strength based on characteristic parameters of FRP-RC beams. The RMSE is used as the fitness value for searching the optimal combination of two hyperparameters of the RF model in the optimizer. The goal is to minimize the fitness value continuously to determine the optimal hyperparameter combination. Figure 7 shows the changes in fitness value curves for the three types of optimization algorithms. Changes have been made to the diagram.

Based on the results in Figure 7, it can be observed that the convergence speed is fastest when the population size is 200 among all ALO-RF models, and the final convergence results of the model are not significantly different when the population size is 50, 100, or 200. For the MFO-RF models, a population size of 100 yields relatively better convergence results. Two SSA-RF models with a population size of 100 or 200 achieved lower final convergence fitness values than other models that showed similar excellent results. Comparing the three hybrid models (ALO-RF, MFO-RF, and SSA-RF), it is found that ALO-RF and MFO-RF have faster convergence speeds than SSA-RF, indicating that ALO and MFO have more sensitive search abilities for the optimal hyperparameters in RF that are suitable for predicting punching shear strength. However, the final convergence fitness values of the three hybrid models are not significantly different, and further analysis is needed to evaluate their performance in predicting the punching shear strength of FRP-RC beams.

### 4.2. Hybrid Models Performance Evaluation

**Figure 8:**
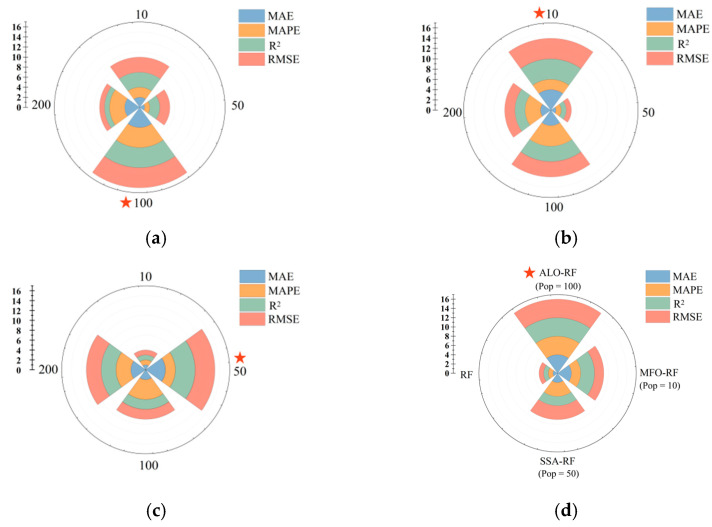
Quantitative comparison of the prediction performance of each model (Red pentagrams indicate optimal models). (**a**) ALO-RF, (**b**) MFO-RF, (**c**) SSA-RF, and (**d**) hybrid models.

To comprehensively evaluate the predictive performance of ALO-RF, MFO-RF, and SSA-RF for PSS, this study calculated four evaluation metrics (MAE, MAPE, R^2^, and RMSE) for each model and scored them based on their ranking in each metric (four points for the best ranking, and one point for the worst ranking), selecting the model with the highest overall score as the current optimal model. The score rankings of each model are shown in Figure 8, with larger fan-shaped areas indicating higher scores and better predictive performance for PSS. As shown in Figure 8a, the evaluation metrics of ALO-RF (Pop = 100) are significantly better than those under other population sizes, indicating that 100 is the optimal population size for ALO-RF to predict PSS in FRP-RC beams. From Figure 8b, it can be seen that MFO-RF (Pop = 10) has a slightly inferior performance in MAPE compared to the models with population sizes of 100 and 200, but its evaluation metrics in MAE, R^2^, and RMSE are superior to other models, and its overall performance is the best among all population sizes. Similarly, Figure 8c shows that SSA-RF (Pop = 50) exhibits the best predictive performance for PSS.

**Figure 9:**
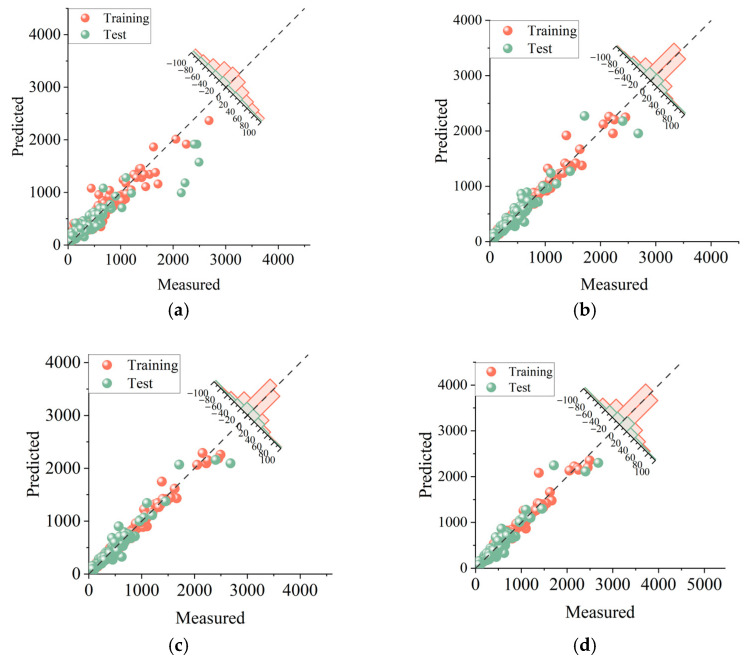
Distribution of results for ALO-RF (Pop = 100), MFO-RF (Pop = 10), SSA-RF (Pop = 50), and unoptimized RF models. (**a**) RF, (**b**) ALO-RF (Pop = 100), (**c**) MFO-RF (Pop = 10), and (**d**) SSA-RF (Pop = 50).

**Figure 10:**
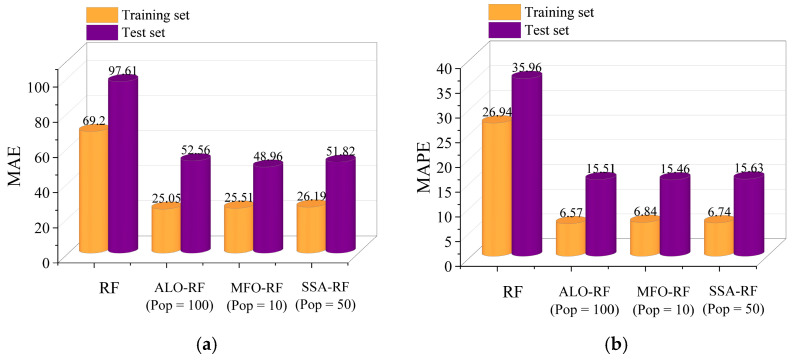

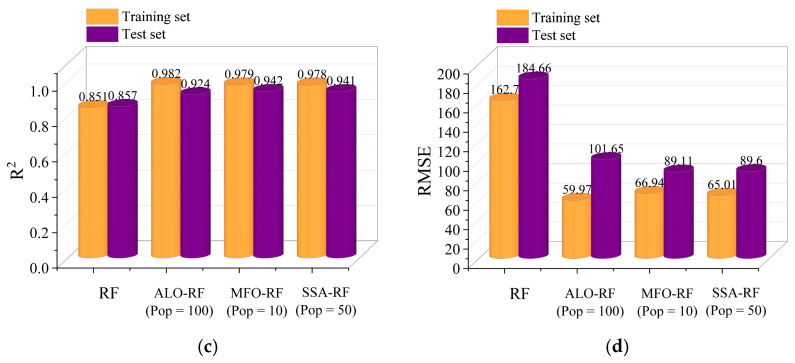
The results for ALO-RF (Pop = 100), MFO-RF (Pop = 10), SSA-RF (Pop = 50), and unoptimized RF models. (**a**) MAE, (**b**) MAPE, (**c**) R^2^, and (**d**) RMSE.

To further compare the PSS prediction performance of ALO-RF (Pop = 100), MFO-RF (Pop = 10), and SSA-RF (Pop = 50), and validate the effectiveness of the three metaheuristic optimization algorithms, this study introduced an unoptimized RF model that was trained and tested on the same dataset with parameters set as shown in Table 5. The comparison between predicted and measured PSS values for FRP-RC beams is visually presented in Figure 9 and Figure 10. In the figure, the red and green dots represent the distribution of predicted and measured values in the training and testing sets, respectively. The closer these points are to the diagonal dashed line, the better the prediction performance of the model. The difference distribution plot in the upper right corner shows the distribution of differences between predicted and measured values. It can be seen from Figure 9 that the error distribution range of ALO-RF (Pop = 100), MFO-RF (Pop = 10), and SSA-RF (Pop = 50) is smaller and more concentrated compared to the unoptimized RF model, indicating that the three metaheuristic optimization algorithms effectively reduce the prediction error. Furthermore, the error distribution of ALO-RF (Pop = 100) is mainly concentrated in the range of [−20, 20], while that of MFO-RF (Pop = 10) is mainly concentrated in the range of [−40, 40], and that of SSA-RF (Pop = 50) is mainly concentrated in the range of [−60, 60], indicating that ALO-RF (Pop = 100) has a smaller error range in predicting the PSS for FRP-RC beams.

**Figure 11:**
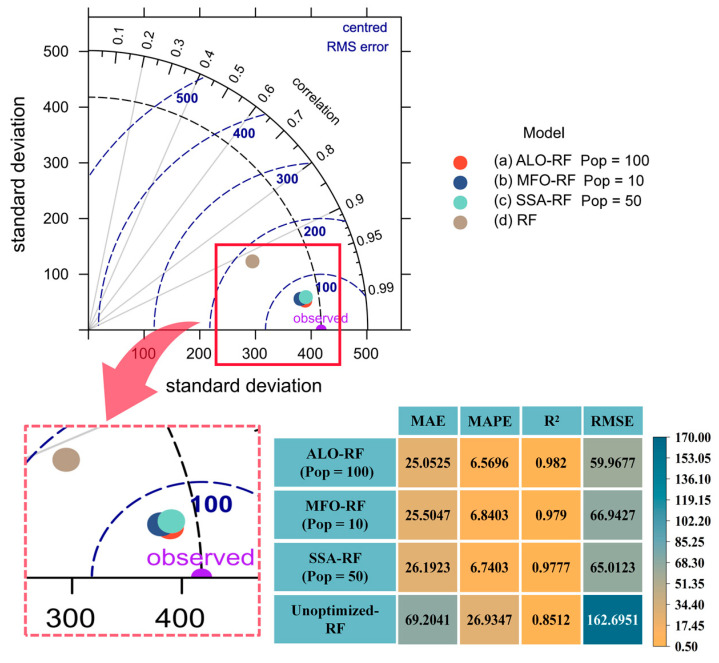
Taylor diagram analysis of the hybrid model (training set).

The Taylor diagram is a graphical tool used to compare multiple models or observations in terms of their correlation, variance, and overall pattern [106]. In Figure 11, Taylor diagrams are used to analyze the performance of ALO-RF (Pop = 100), MFO-RF (Pop = 10), SSA-RF (Pop = 50), and unoptimized RF in predicting the PSS of FRP-RC beams. As shown in Figure 10a, the data points of ALO-RF (Pop = 100), MFO-RF (Pop = 10), and SSA-RF (Pop = 50) in the training set are closer to the observation points than RF, indicating smaller RMSE values. ALO-RF (Pop = 100) also has a smaller azimuth angle, indicating a higher correlation coefficient. These findings suggest that ALO, MFO, and SSA optimization algorithms can efficiently capture the inherent relationship between various characteristic parameters of FRP-RC beams and PSS, and search for suitable hyperparameter combinations to improve the predictive performance of the RF model.

Based on the comprehensive analysis, and combined with Figure 8d, ALO-RF (Pop = 100) demonstrated the best predictive performance, with MAE of 25.0525, MAPE of 6.5696, R^2^ of 0.9820, and RMSE of 59.9677 in the training set, and MAE of 52.5601, MAPE of 15.5083, R^2^ of 0.941, and RMSE of 101.6494 in the testing set. These results indicate that the ALO algorithm with a population size of 100 can effectively capture the optimal hyperparameter combination and further improve the ability of the RF model to capture the intrinsic relationship between the input parameters and PSS of FRP-RC beam.

Feng et al. [20] developed various ML algorithms and compared their predictive performance for assessing the PSS of FRP-RC beams. However, these models were confined to traditional, independent ML techniques. In contrast, the present research employs a RF model with an enhanced ability to prevent overfitting. Additionally, three metaheuristic optimization algorithms were introduced to tune the hyperparameter of the RF model, enabling better identification of the intrinsic relationship between input parameters of FRP-RC beams and the PSS, thereby improving prediction accuracy.

To validate the effectiveness and superiority of the proposed predictive model, the best prediction performance of the ALO-RF model was compared with other models developed using the same database. The performance comparison results of the three models are listed in Table 6. The results show that the proposed ALO-RF model has higher prediction performance than previously published models by means of the higher R^2^ values. This suggests that the ALO-RF model provides a better explanation of the relationship between input parameters and the PSS of FRP-RC beams. The methodology can be adapted and fine-tuned to address specific challenges or research questions in different areas, leading to more accurate and reliable predictions [107,108]. The methodology can be adapted and fine-tuned to address specific challenges or research questions in different areas, leading to more accurate and reliable predictions.

### 4.3. Importance Evaluation of Feature Parameters

The feature importance analysis is an essential step in understanding the underlying relationships between the input features and the target variable, which can be used to improve the performance and interpretability of the model. RF can provide important insights into the relative importance of different features in predicting the target variable. This approach involves a quantitative assessment of the contribution of each feature to the predictive performance of the model, enabling researchers to identify the most significant features for the task at hand [109,110,111,112,113,114,115,116,117,118,119]. These importance scores can be calculated by the Gini index:

(7) $$\begin{array}{cc} Gini(X_{a}) & =\sum_{b-1}^{B}P(X_{a}=Y_{b})(1-P(X_{a}=Y_{b}))\\ & =1-\sum_{b}^{B}P{\left(X_{a}=Y_{b}\right)}^{2} \end{array}$$

where *Gini*(*X_a_*) represents the Gini index, *P*(*X_a_* = *Y_b_*) represents probability, and *X_a_* = *Y_b_* represents an estimated value.

**Figure 12:**
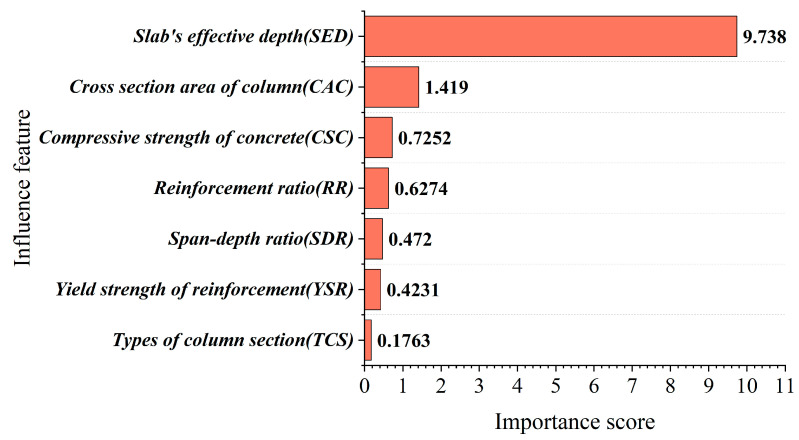
Importance analysis of input parameters of FRP-RC beams.

Figure 12 displays the importance scores of each input variable in the RF model, and according to the data in the figure, it can be seen that slab’s effective depth (*SED*) has the highest importance score among all the feature parameters of FRP-RC beams. The result highlights the close relationship between SED and PSS, which plays a prominent role in predicting the PSS based on the ALO-RF model. Although the importance scores of CAC, CSC, RR, SDR, YSR, and TCS are lower compared to SED, they still have a significant underlying relationship with PSS. Overall, slab’s effective depth (SED) has the highest contribution to predicting the PSS among all the parameters, indicating a strong internal relationship between SED and PSS, and adjusting SED can effectively control the variation in PSS.

## 5. Conclusions

This study investigates the efficacy of three metaheuristic optimization algorithms (ALO, MFO, and SSA) in fine-tuning the hyperparameters of a random forest (RF) model for predicting the PSS of FRP-RC beams. The results demonstrate that the ALO-RF model exhibits a faster convergence rate compared to both MFO-RF and SSA-RF, indicating that ALO and MFO exhibit heightened sensitivity in searching for optimal hyperparameters when predicting the PSS of FRP-RC beams using the RF model. Notably, the ALO-RF model, employing a population size of 100, yields the most accurate predictive performance, characterized by a narrower distribution range of errors and a higher correlation coefficient. Furthermore, the study identifies the significant energy dissipation (SED) as the input parameter exerting the most significant influence on PSS prediction, emphasizing that adjusting the SED can effectively regulate PSS variations.

However, the scarcity of experimental data presents a significant challenge to accurately predicting the performance of FRP-RC beams, which in turn hampers the development and validation of predictive models. Furthermore, the intricate material behavior between the FRP reinforcement and the concrete matrix adds to the complexity of prediction efforts. Although advanced machine learning and optimization algorithms can provide accurate predictions, they often involve high computational requirements, which can result in prolonged training and prediction times. In future research, increasing the amount of experimental data on FRP-RC beams may improve the quality and generalizability of predictive models. Additionally, enhancing our understanding of the material behavior between the FRP reinforcement and the concrete matrix can aid in the development of more precise and dependable prediction models.

## Figures and Tables

**Table 1:** Statistical information of parameters included in the FRP-RC beams database.

Category	Parameter	Unit	Min.	Max.	Mean	Type
Geometric parameters	Types of column section (TCS)	-	1	3	-	Input
	Cross section area of column (CAC)	cm^2^	20.43	6375.87	489.59	Input
	Slab’s effective depth (SED)	mm	29.97	668.50	113.88	Input
	Span–depth ratio (SDR)	mm	0.61	32.51	6.60	Input
Concrete strength information	Compressive strength of concrete (CSC)	MPa	9.40	130.10	35.42	Input
Steel reinforcement information	Yield strength of reinforcement (YSR)	MPa	234.70	749.00	456.61	Input
	Reinforcement ratio (RR)	%	0.25	3.00	1.23	Input
	Punching shear strength (PSS)	kN	24.00	4915.00	403.77	Output

Note: Min. = minimum; Max. = maximum.

**Table 2:** Punching shear strength database for FRP-RC beams (partial data only).

No.	TCS	CAC (cm^2^)	SED (mm)	SDR (mm)	CSC (MPa)	YSR (MPa)	RR (%)	PSS (kN)
1	1	2580.64	125.48	7.09	35.40	331.00	1.52	579.00
2	1	625.00	125.00	3.80	19.12	291.20	1.21	446.00
3	1	625.00	57.00	8.33	16.83	282.40	0.99	82.50
4	1	256.00	80.00	10.44	25.83	525.00	1.31	190.00
5	1	900.00	170.00	3.53	44.00	343.00	0.79	660.00
6	1	625.00	127.50	6.20	70.00	433.00	2.68	801.00
7	3	355.11	91.00	8.06	19.80	352.00	1.58	252.00
8	1	225.00	95.00	7.11	67.00	490.00	0.49	178.00
9	3	288.00	109.00	8.53	58.00	749.00	1.07	246.00
10	3	400.00	150.00	7.50	22.59	453.60	0.86	498.20
11	2	383.60	82.00	5.16	16.99	471.00	0.53	152.00
12	2	176.71	100.00	6.11	55.70	650.00	0.87	341.00
13	1	625.00	119.00	3.99	28.44	256.60	1.60	600.00
14	1	404.01	78.00	8.97	10.82	403.00	0.66	128.00
15	1	625.00	80.00	5.94	50.86	289.10	1.32	302.40
16	1	625.00	150.00	5.00	36.04	604.00	1.28	855.00
17	1	625.00	121.00	3.93	19.36	271.80	1.42	515.00
18	1	104.04	60.80	6.27	22.36	376.00	3.14	105.00
…	…	…	…	…	…	…	…	…

**Table 3:** Definition and calculation of evaluation indicators.

Indicator	Definition	Calculation formula
MAE	The average absolute difference between the predicted values and the measured values.	$\mathrm{MAE}=\frac{1}{m}\sum_{i=1}^{m}\left|{\hat{y}}_{i}-y_{i}\right|$
MAPE	The average percentage difference between the predicted values and the measured values.	$\mathrm{MAPE}=\frac{100\%}{m}\sum_{i=1}^{m}\left|\frac{{\hat{y}}_{i}-y_{i}}{y_{i}}\right|$
R^2^	The statistical measure that represents the proportion of the variance in the target variable.	$R^{2}=1-\frac{\sum_{i=1}^{m}{\left({\hat{y}}_{i}-y_{i}\right)}^{2}}{\sum_{i=1}^{m}{\left({\bar{y}}_{i}-y_{i}\right)}^{2}}$
RMSE	The root mean squared difference between the predicted values and the measured values.	$\mathrm{RMSE}=\sqrt{\frac{1}{m}\sum_{i=1}^{m}{\left({\hat{y}}_{i}-y_{i}\right)}^{2}}$

Note: ${\hat{y}}_{i}$ represents the predicted value; ${\bar{y}}_{i}$ represents the average value; $y_{i}$ represents the measured value; and *m* is the training or testing samples.

**Table 4:** Parameter setting of the hybrid models.

Hybrid Model	Number of Iterations	Population Size	Objectives of Optimization
ALO-RF	600	10, 50, 100, 200	The number of trees. The maximum depth of trees.
MFO-RF	600	10, 50, 100, 200	
SSA-RF	600	10, 50, 100, 200	

**Table 5:** Parameter setting of RF model.

Model	Hyperparameter	Typical Default Values	Reference
RF	mtry	*v*/3 for regression	[105]
	samples size	n	
	node size	5 (for regression)	
	number of trees	1000	
	splitting rule	Gini impurity	

Note: n is the number of observations and v is the number of variables in the dataset.

**Table 6:** Performance comparison between previous works and the proposed model.

Reference	Model	Performance (R^2^)
Feng et al. [20]	ANN	0.856
	SVM	0.852
	DT	0.887
	AdaBoost	0.919
	XGBoost	0.928
This paper	ALO-RF	0.941

Note: ANN—Artificial neural network; SVM—support vector machine; DT—decision trees; adaboost—adaptive boosting; and XGBoost—eXtreme gradient boosting.

## Data Availability

The authors do not have permission to share data.
